# A Cooperative Model for IS Security Risk Management in Distributed Environment

**DOI:** 10.1155/2014/167497

**Published:** 2014-01-19

**Authors:** Nan Feng, Chundong Zheng

**Affiliations:** College of Management and Economics, Tianjin University, 92 Weijin Road, Nankai District, Tianjin 300072, China

## Abstract

Given the increasing cooperation between organizations, the flexible exchange of security information across the allied organizations is critical to effectively manage information systems (IS) security in a distributed environment. In this paper, we develop a cooperative model for IS security risk management in a distributed environment. In the proposed model, the exchange of security information among the interconnected IS under distributed environment is supported by Bayesian networks (BNs). In addition, for an organization's IS, a BN is utilized to represent its security environment and dynamically predict its security risk level, by which the security manager can select an optimal action to safeguard the firm's information resources. The actual case studied illustrates the cooperative model presented in this paper and how it can be exploited to manage the distributed IS security risk effectively.

## 1. Introduction

With the increasing of collaboration between organizations, the management of information systems (IS) security risk is distributed across the allied organizations and the cooperative activities between organizations are imperative [1–4]. Therefore, for more effectively assessing the security risk level of the IS in a distributed environment, it is critical to develop a system for the exchange of security information among the interconnected IS. However, how to achieve the flexible exchange of security information under distributed environment is a significant challenge in the process of modelling [5]. Unfortunately, few previous studies on IS security take the above issue into account.

In this paper, a cooperative model for IS security risk management is proposed to estimate the risk level of each associated organization's IS and support the decision making of security risk treatment in a distributed environment. In the model, the exchange of security information among the interconnected IS is achieved through Bayesian networks (BNs). Moreover, a BN is also exploited to model the security environment of an organization's IS and predict its security risk level. However, it is difficult and critical task for a security manager to establish an appropriate BN, which is suitable for the environment of organization's information systems [6–8]. To address this issue, in this paper, we develop an algorithm to support the BN initiation. Finally, based on the security risk level for an organization's IS, the security manager selects an optimal action to protect its information resources.

The remaining sections of this paper are organized as follows. We first review the relevant literature in Section 2. Then we discuss the development of the cooperative model in detail in Sections 3 and 4. The proposed model is further demonstrated and validated in Section 5 via a case study. Finally, we summarize our contributions and point out further research directions.

## 2. Literature Review

There has been increased academic interest in the IS security risk management. From the technical literature, the security protocols [9], fire wall and intrusion detection techniques [10, 11], and authentication technologies [12, 13] have been examined. From an economics perspective, some researchers have investigated the investment on information systems security [14, 15], economics of vulnerability disclosure [16, 17], and the characteristics of internet security breaches that impact the market value of breached firms [18].

In recent years, a new managerial perspective on IS security has emerged from the literature. This perspective focuses on the managerial processes that control the effective deployment of technical approaches and security resources to create a secure IS environment in an organization. From this perspective, Feng and Li [19] proposed an IS security risk evaluation model based on the improved evidence theory. For the handling of uncertain evidence found in IS security risk analysis, their model provided a novel approach to define the basic belief assignment of evidence theory. In addition, the model also presented a method of testing the evidential consistency, which is capable of resolving the conflicts from uncertain evidence. Then, in order to identify the causal relationships among security risk factors and analyze the complexity of vulnerability propagation, they also developed a security risk analysis model (SRAM) [20], in which the vulnerability propagation analysis is performed to determine the propagation paths with the highest IS security risk level. Yan [21] presented a conceptual model for IS security analysis, which can facilitate to identify potential security risks. Chen et al. [22] focus on controlling the risks in the form of the fault of information networks. They developed an approach to estimate the risk level on the vulnerability of information networks.

Bayesian networks (BNs), also known as probabilistic belief networks, is a knowledge representation tool capable of representing dependence and independence relationships among random variables [23]. A BN, *N* = (*X*, *G*, *P*), consists of a directed acyclic graph *G* and a set of conditional probability distributions (beliefs) *P* for variables *X*. BN inference means computing the conditional probability for some variables given the evidence, which is defined as a collection of findings. This operation is also called probability updating or belief updating.

In this paper, the developed BN is not only used to facilitate the dynamical prediction of the security risk level of an organization's IS, but also exploited to model the IS security environment.

## 3. Model Architecture

**Figure 1:**
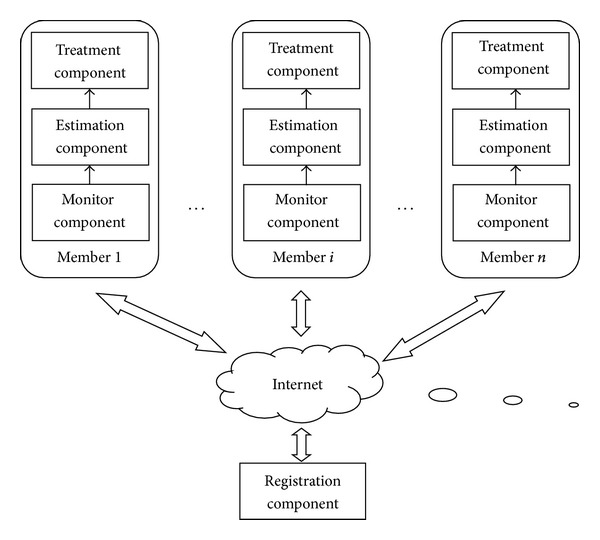
Model architecture.

In a distributed environment, the proposed model consists of many interconnected network information systems. We call these network information systems as “associated members.” Each associated member is installed with three kinds of components: monitor component, estimation component, and treatment component. Besides, the above three kinds of components, the registration component contains the information about each estimation component. It is required that all estimation components in the distributed environment must register with the registration component. The cooperative model architecture is demonstrated in Figure 1.

**Figure 2:**
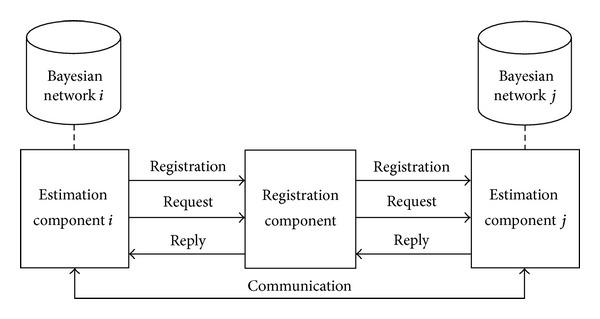
Interactions among the components.

The interactions among the estimation component and the registration component are shown in Figure 2. In the interactive process, as shown in Table 1, there are four kinds of sharing information: search request, search reply, registration message, and communication between estimation components.

## 4. Bayesian Network Development

In this section, we present an algorithm based on ant colony optimization (shown in Algorithm 1) to develop the Bayesian network (BN), which is able to model the security environment of an associated member under distributed environment.

The equations appearing in the algorithm are as follows.

(1) Heuristic information:

(1) $$\begin{array}{c} \eta_{ij}=f\left(x_{i},Pa\left(x_{i}\right)\cup \left\{x_{j}\right\}\right)-f\left(x_{i},Pa\left(x_{i}\right)\right). \end{array}$$

(2) Updating rule:

(2) $$\begin{array}{c} \tau_{ij}⟵\left(1-\rho \right)\tau_{ij}+\rho \Delta \tau_{ij} \end{array}$$

in which

(3) $$\begin{array}{c} \Delta \tau_{ij}=\{\begin{array}{cc} \frac{1}{\left|f\left(G^{*}:D\right)\right|} & \text{if}\;x_{j}⟶x_{i}\in G^{*}\\ \tau_{ij} & \text{if}\;x_{j}⟶x_{i}\notin G^{*}, \end{array} \end{array}$$

in the arc *x*_*j*_ → *x*_*i*_, *τ*_*ij*_ is the pheromone's degree, and *ρ*  (0 < *ρ* ≤ 1) is a variable which can control the pheromone value. Moreover, G* is the BN structure suitable for the organization's IS best.

(3) Probabilistic transition:

(4) $$\begin{array}{c} r,l=\{\begin{array}{cc} \underset{i,j\in F_{G}}{\arg\;\max}\left\{{\left[\tau_{ij}\right]}^{\alpha }{\left[\eta_{ij}\right]}^{\beta }\right\} & \text{if}\;q\leq q_{0}\\ I,J & \text{if}\;q>q_{0} \end{array} \end{array}$$

in which *I* and *J* are two nodes chosen randomly based on the following equation:

(5) $$\begin{array}{c} p_{k}\left(i,j\right)=\{\begin{array}{cc} \frac{{\left[\tau_{ij}\right]}^{\alpha }{\left[\eta_{ij}\right]}^{\beta }}{\sum_{u,v\in F_{G}}^{}{\left[\tau_{uv}\right]}^{\alpha }{\left[\eta_{uv}\right]}^{\beta }} & \text{if}\;i,j\in F_{G}\\ 0 & \text{otherwise}. \end{array} \end{array}$$

## 5. Case Study

In this section, the proposed model is applied to a distributed environment, which is composed of four associated members with interconnected IS: two suppliers (S1 and S2), a manufacturer (M1), and a downstream transporter (DT1).

**Figure 3:**
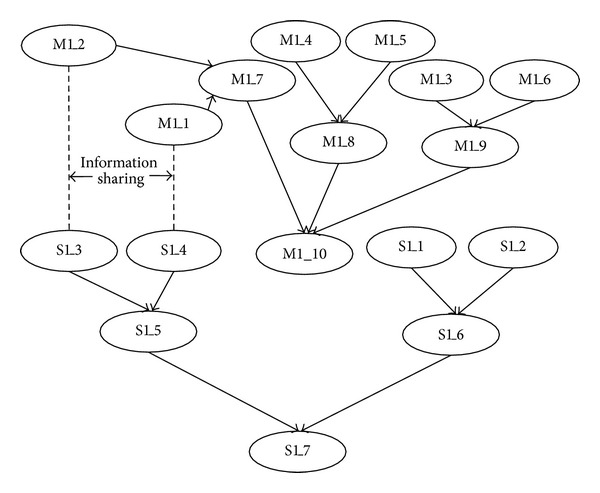
BN structures of M1 and S1.

Based on the algorithm presented in Section 4, we develop the BN for each associated member. Taking M1 and S1, for example, their information of BN nodes is given in Tables 2 and 3, and their BN structures are shown in Figure 3.

**Figure 4:**
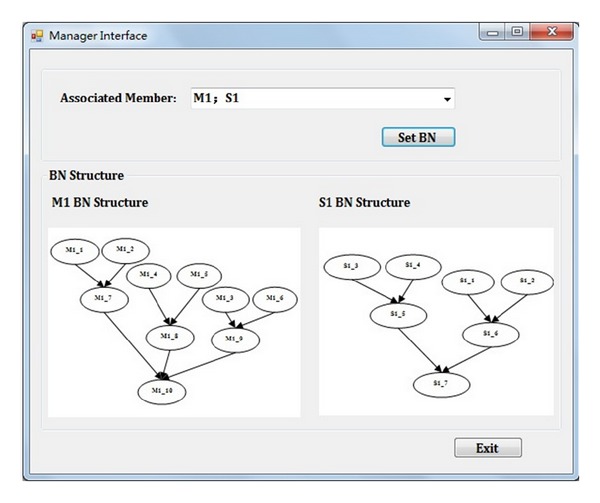
Security manager interface.

The manager interface of our proposed model is shown in Figure 4, in which the security manager can specify the BN for each associated organization.

Once the new evidence is obtained through the monitor components, the estimation component is able to make the BN modify its own belief (probability distribution on variable of risk level) in real time and exchange the update of beliefs of the security state with other associated members.

## 6. Conclusions

In a distributed environment, in order to effectively manage information systems (IS) security, a cooperative model based on Bayesian networks is presented and illustrated in this paper. We contribute to the IS security literature by supporting the exchange of security information among interconnected IS. Furthermore, for the modelling of IS security environment, an algorithm based on ant colony optimization facilitates to predict IS threat level more objectively. The model proposed in this paper has great potential for future extensions and refinements to provide more utility for the management of IS security.

## Figures and Tables

**Algorithm 1:**
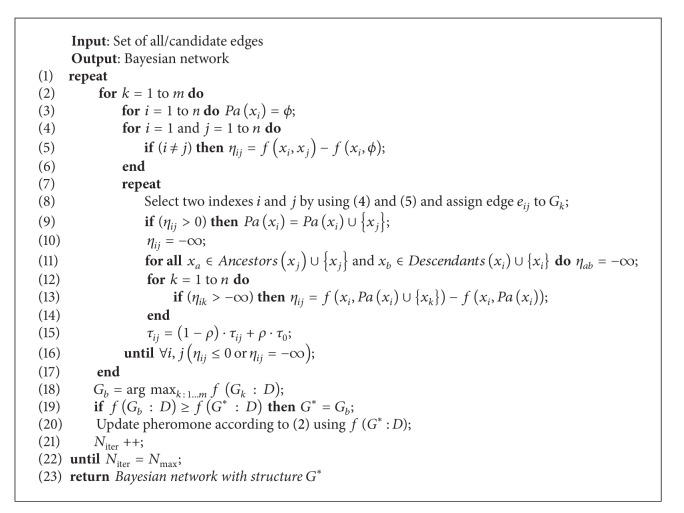
Bayesian network development algorithm.

**Table 1:** Information exchange in the interactive process.

Exchange information	Description
Search request	It consists of the requester's id, IP address, and the required input variables. The estimation component has a set of sharing variables. To find components capable of providing required input data, the estimation component sends a search request to the registration component.
Search reply	It consists of the requested variable name, the IP address, and status of the component publishing the variable. Based on receiving a search request, the registration component searches its database to determine which components can provide the requested variables.
Registration message	It consists of component id, IP address, list of published variables, and their possible states. Each estimation component registers with the registration component, which issues an acknowledgment message for entering the new component in its database.
Communication between estimation components	It consists of the request id, the sender's id, and the probability distribution of the requested variable. Upon receiving the list of components capable of providing the required input from the registration component, the request component sends requests directly to these components. Then, the sender sends the probability distribution of the requested variable.

**Table 2:** BN information of M1.

Node ID	Node name	State space	Parent nodes	Children nodes
M1_1	Network access control	{Effective; average; ineffective}	Φ	{M1_7}
M1_2	Network security audit	{Comprehensive; incomprehensive}	Φ	{M1_7}
M1_3	Change management	{Effective; average; ineffective}	Φ	{M1_9}
M1_4	Supplier threat level	{0; 1; 2; 3; 4; 5}	Φ	{M1_8}
M1_5	Transporter threat level	{0; 1; 2; 3; 4; 5}	Φ	{M1_8}
M1_6	Operational procedures and responsibilities	{Very standard; standard; non-standard}	Φ	{M1_9}
M1_7	Network security	{High; medium; low}	{M1_1, M1_2}	{M1_10}
M1_8	External systems security	{High; medium; low}	{M1_4, M1_5}	{M1_10}
M1_9	Operation security	{High; medium; low}	{M1_3, M1_6}	{M1_10}
M1_10	M1 threat level	{0; 1; 2; 3; 4; 5}	{M1_7, M1_8, M1_9}	Φ

**Table 3:** BN information of S1.

Node ID	Node name	State space	Parent nodes	Children nodes
S1_1	Communication secrecy	{High; medium; low}	Φ	{S1_6}
S1_2	Audit logging	{Secure; average; insecure}	Φ	{S1_6}
S1_3	Network access control	{Effective; average; ineffective}	Φ	{S1_5}
S1_4	Network security audit	{Comprehensive; incomprehensive}	Φ	{S1_5}
S1_5	Network security	{High; medium; low}	{S1_3, S1_4}	{S1_7}
S1_6	Communication security	{High; medium; low}	{S1_1, S1_2}	{S1_7}
S1_7	S1 threat level	{0; 1; 2; 3; 4; 5}	{S1_5, S1_6}	Φ

## References

[1] Tsoukalas IA, Siozos PD (2011). Privacy and anonymity in the information society—challenges for the european union. *TheScientificWorldJournal*.

[2] Zhang Y, Deng X, Wei D, Deng Y (2012). Assessment of E-Commerce security using AHP and evidential reasoning. *Expert Systems with Applications*.

[3] Ransbotham S, Mitra S (2009). Choice and chance: a conceptual model of paths to information security compromise. *Information Systems Research*.

[4] Bulgurcu B, Cavusoglu H, Benbasat I (2010). Information security policy compliance: an empirical study of rationality-based beliefs and information security awareness. *MIS Quarterly*.

[5] Gal-Or E, Chose A (2005). The economic incentives for sharing security information. *Information Systems Research*.

[6] Fan C-F, Yu Y-C (2004). BBN-based software project risk management. *Journal of Systems and Software*.

[7] Sun L, Srivastava RP, Mock TJ (2006). An information systems security risk assessment model under the Dempster-Shafer theory of belief functions. *Journal of Management Information Systems*.

[8] Yue WT, Çakanyildirim M, Ryu YU, Liu D (2007). Network externalities, layered protection and IT security risk management. *Decision Support Systems*.

[9] Di Pietro R, Mancini LV (2003). Security and privacy issues of handheld and wearable wireless devices. *Communications of the ACM*.

[10] Ning P, Cui Y, Reeves DS, Xu D (2004). Techniques and tools for analyzing intrusion alerts. *ACM Transactions on Information and System Security*.

[11] Sarathy R, Muralidhar K (2002). The security of confidential numerical data in databases. *Information Systems Research*.

[12] Li N, Tripunitara MV (2006). Security analysis in role-based access control. *ACM Transactions on Information and System Security*.

[13] Rinderle-Ma S, Reichert M (2009). Comprehensive life cycle support for access rules in information systems: the CEOSIS project. *Enterprise Information Systems*.

[14] Gordon LA, Loeb MP (2002). The economics of information security investment. *ACM Transactions on Information and System Security*.

[15] Herath HSB, Herath TC (2009). Investments in information security: a real options perspective with Bayesian postaudit. *Journal of Management Information Systems*.

[16] Kannan K, Telang R (2005). Market for software vulnerabilities? Think again. *Management Science*.

[17] Azaiez MN, Bier VM (2007). Optimal resource allocation for security in reliability systems. *European Journal of Operational Research*.

[18] Cavusoglu H, Mishra B, Raghunathan S (2004). The effect of internet security breach announcements on market value: capital market reactions for breached firms and internet security developers. *International Journal of Electronic Commerce*.

[19] Feng N, Li M (2011). An information systems security risk assessment model under uncertain environment. *Applied Soft Computing Journal*.

[20] Feng N, Wang HJ, Li M (2014). A security risk analysis model for information systems: causal relationships of risk factors and vulnerability propagation analysis. *Information Sciences*.

[21] Yan Q (2008). A security evaluation approach for information systems in telecommunication enterprises. *Enterprise Information Systems*.

[22] Chen P-Y, Kataria G, Krishnan R (2011). Correlated failures, diversification, and information security risk management. *MIS Quarterly*.

[23] Pearl J (1998). *Probabilistic Reasoning in Intelligent Systems: Networks of Plausible Inference*.

